# Botulinum neurotoxin A ameliorates depressive-like behavior in a reserpine-induced Parkinson’s disease mouse model via suppressing hippocampal microglial engulfment and neuroinflammation

**DOI:** 10.1038/s41401-023-01058-x

**Published:** 2023-02-10

**Authors:** Yang Li, Qiao Yin, Qi Li, An-ran Huo, Ting-ting Shen, Jia-qian Cao, Chun-feng Liu, Tong Liu, Wei-feng Luo, Qi-fei Cong

**Affiliations:** 1 Department of Neurology and Clinical Research Center of Neurological Disease, The Second Affiliated Hospital of Soochow University, Suzhou, 215004 China; 2 Department of Neurology, Huzhou Central Hospital, The Affiliated Huzhou Hospital, Zhejiang University School of Medicine, Huzhou, 313000 China; 3 Institute of Neuroscience and Jiangsu Key Laboratory of Neuropsychiatric Diseases, Soochow University, Suzhou, 215123 China; 4 Institute of Pain Medicine and Special Environmental Medicine, Nantong University, Nantong, 226019 China

**Keywords:** Parkinson’s disease, depression, reserpine, botulinum neurotoxin A, microglia, complement

## Abstract

Depression is one of the common non-motor symptoms of Parkinson’s disease (PD). In the clinic, botulinum neurotoxin A (BoNT/A) has been used to treat depression. In this study, we investigated the mechanisms underlying the anti-depressive effect of BoNT/A in a PD mouse model. Mice were administered reserpine (3 μg/mL in the drinking water) for 10 weeks. From the 10^th^ week, BoNT/A (10 U·kg^−1^·d^−1^) was injected into the cheek for 3 consecutive days. We showed that chronic administration of reserpine produced the behavioral phenotypes of depression and neurochemical changes in the substantia nigra pars compacta (SNpc) and striatum. BoNT/A treatment significantly ameliorated the depressive-like behaviors, but did not improve TH activity in SNpc of reserpine-treated mice. We demonstrated that BoNT/A treatment reversed reserpine-induced complement and microglia activation in the hippocampal CA1 region. Furthermore, BoNT/A treatment significantly attenuated the microglial engulfment of presynaptic synapses, thus ameliorating the apparent synapse and spine loss in the hippocampus in the reserpine-treated mice. Moreover, BoNT/A treatment suppressed microglia-mediated expression of pro-inflammatory cytokines TNF-α and IL-1β in reserpine-treated mice. In addition, we showed that BoNT/A (0.1 U/mL) ameliorated reserpine-induced complement and microglia activation in mouse BV2 microglial cells in vitro. We conclude that BoNT/A ameliorates depressive-like behavior in a reserpine-induced PD mouse model through reversing the synapse loss mediated by classical complement induced-microglial engulfment as well as alleviating microglia-mediated proinflammatory responses.

**Figure:**
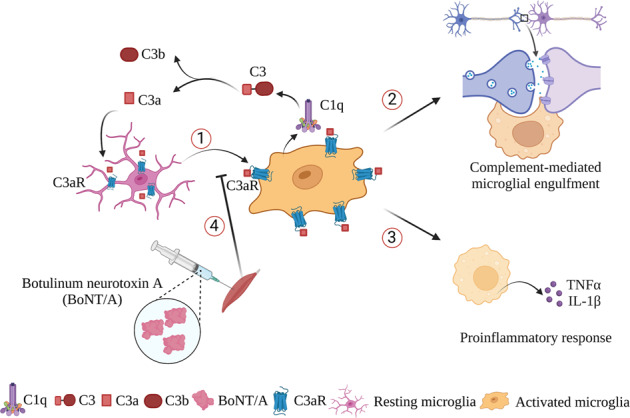
BoNT/A ameliorates depressive-like behavior, and reverses synapse loss mediated by classical complement pathway-initiated microglia engulfment as well as alleviates microglia-mediated proinflammatory response in the reserpine-induced Parkinson’s disease mouse model.

BoNT/A ameliorates depressive-like behavior, and reverses synapse loss mediated by classical complement pathway-initiated microglia engulfment as well as alleviates microglia-mediated proinflammatory response in the reserpine-induced Parkinson’s disease mouse model.

## Highlights

- Botulinum neurotoxin A ameliorates reserpine-induced depressive behaviors.
- Complement activation is involved in the reserpine-induced mouse model.
- Botulinum neurotoxin A reverses spine loss and reduced synapse density.
- Botulinum neurotoxin A inhibits complement activation and microglial engulfment.
- Botulinum neurotoxin A alleviates reserpine-induced neuroinflammation.

## Introduction

Parkinson’s disease (PD) is the second most common neurodegenerative disorder and affects more than 1% of the elderly population [1]. Depression is the most common nonmotor symptom of Parkinson’s disease and may occur at any stage in the course of Parkinson’s disease [2]. Core depressive symptoms include depressive mood, anhedonia, and feelings of worthlessness in patients with depression in PD. The diagnosis of depression is quite challenging because many of its features, such as insomnia, fatigue, loss of appetite, and psychomotor retardation, overlap with the primary symptoms of Parkinson’s disease [3]. These overlaps in symptoms may result in underdiagnosis and poor treatment, thus seriously affecting the quality of life in patients with Parkinson’s disease.

The underlying pathological mechanisms of depression in PD remain elusive. Currently, genetic, epigenetic, and environmental factors are the most studied factors for depression in elderly people. Various alterations in the brain might have impacts on late-life depression, including monoaminergic disturbances, functional damage in the limbic and subcortical neural circuits, hippocampal atrophy, alterations in neurogenesis, and neuroinflammation [4]. The frontotemporal regions, such as the thalamus, amygdala, medial prefrontal cortex, hippocampus, and anterior cingulate cortex, have been identified as potentially relevant brain regions for depression [5]. The degree of hippocampal atrophy is used to predict the severity of mental symptoms in PD patients [6]. There is converging evidence that neuroinflammation and neurotrophic factors are involved in the pathophysiology of depression in Parkinson’s disease [4]. Studies of the roles of inflammatory cytokines in the nonmotor symptoms of PD have found that TNF-α, but not IL-1β, IL-6, or IL-10, was significantly associated with cognition, depression, and disability [7, 8]. Prolonged stress-induced neuroinflammation might cause negative effects on synaptic plasticity and synaptic function. In genetic animal models and human postmortem studies of depression, reductions in synapse number and decreased levels of synaptic signaling proteins are observed [9]. Synapse loss is a common pathological manifestation of multiple neurological diseases. The hippocampus is a temporal lobe structure implicated in depression and the memory process [10, 11]. Hippocampal MRI findings indicate that overall changes specifically in the hippocampal CA1 region are more pronounced in major depressive disorder. Changes in hippocampal CA1-CA3 and dentate gyrus volume are observed in nonmedicated, but not medicated, patients with depression [12]. Moreover, reduced hippocampal CA2-CA3 subfield volume has been found to be related to depression in nonmedicated PD patients [13]. It has been reported that hippocampal dopaminergic dysfunction and serotoninergic dysfunction play a critical role in depression [14]. Both of these monoamines are important neurotransmitters in synaptic transmission. The disruption of synaptogenesis and loss of connections in the hippocampus in depression in PD have attracted increasing interest. However, whether neuroinflammation-mediated synaptic function is involved in depression in PD remains to be explored.

Microglia are the resident immune cells in the central nervous system. During development, microglia are responsible for the survival and apoptosis of neurons, the growth of axons, the migration of neurons, the pruning of supernumerary synapses, and the modulation of the strength of synaptic transmissions [15, 16]. During brain injury, microglia are known to phagocytose particular antigens, protein aggregates, and dead cells, as well as inflammatory factors [17]. Postmortem studies have demonstrated microglial activation in the hippocampus in depression in PD [18]. Chronic impairment in microglia-neuron interactions may secure the permanence of the failure of synaptic and neuronal function and health in Parkinson’s disease. The involvement of microglia and complement in neurological disorders has been attributed to neuroinflammation [19], as well as complement-mediated microglial synaptic engulfment [20]. In recent years, emerging data have demonstrated that the complement proteins C1q and C3 and their regulators are involved in synapse elimination during neurodevelopment [21, 22] and synapse loss in the neurodegeneration process [23, 24]. Selective overexpression of complement C3 in the medial prefrontal cortex (mPFC) was sufficient to produce depression-like behavior in mice, while C3 and C3aR knockout mice were resilient to chronic stress-induced depressive-like behavior [25]. Moreover, it has been shown that the mice of an established C1q knockout depression model had reduced synapse loss in the amygdala and reduced depression-like behavior, suggesting a key role for C1q in the pathogenesis of depression [26]. Nevertheless, how complement-mediated microglial engulfment impacts synaptic dysfunction in depression in Parkinson’s disease remains unknown. Thus, we wanted to determine whether complement-mediated microglial synaptic engulfment is functionally involved in depression in PD.

Botulinum neurotoxin A (BoNT/A) is a metalloprotease produced by the bacterium *Clostridium botulinum* and yields a sustained blockade of acetylcholine release from peripheral nerve terminals, thus reducing muscle hyperactivity to relieve pain with a long-lasting therapeutic effect [27]. Recently, a few clinical studies and randomized controlled trials investigated the beneficial role of BoNT/A in the management of major depressive disorders. BoNT/A produced significant improvement in depressive symptoms and acted as a safe and sustainable treatment for patients with depression [28, 29]. Emerging evidence has shown that alterations in the brain may result from either sensory input from the peripheral nervous system (PNS) via retrograde transport of BoNT/A or direct injection of BoNT/A into the central nervous system (CNS) [30]. Our previous study revealed that BoNT/A could alleviate depressive symptoms in chronic restraint stress-induced mice via an increase in 5-HT levels and the activation of the BDNF/ERK/CREB pathways [31]. Currently ongoing studies for BoNT/A are exploring novel indications and associated mechanisms underlying depression in PD. In a clinical study, BoNT/A ameliorated depressive symptoms rapidly and effectively in patients with Parkinson’s disease [32]. Our previous clinical studies demonstrated that BoNT/A treatment had effects that are similar to those of sertraline on parkinsonism depression [33]. BoNT/A improved the symptoms of dyskinesia in the MPTP and 6-OHDA-induced Parkinson’s disease rodent animal models [34] and alleviated depression-like behavior in a unilateral 6-OHDA rat model of Parkinson’s disease [35]. However, the underlying mechanism of action of BoNT/A in the treatment of parkinsonism depression remains unclear.

Therefore, in the present study, we sought to explore the antidepressant effects and underlying mechanisms of BoNT/A on reserpine-induced depression in an established Parkinson’s disease mouse model. BoNT/A was administered as a single cheek injection, and its efficacy in the treatment of antidepressant behavior in the Parkinson’s disease mouse model was evaluated. We mainly focused on the effects of BoNT/A treatment on complement-mediated microglial synaptic engulfment and microglia-mediated neuroinflammation in the process of depression in Parkinson’s disease.

## Materials and methods

### Animals

Adult (approximately 30 g, 6–8 weeks old) male ICR mice were purchased from the Shanghai SLAC Laboratory Animal Co., Ltd. (Shanghai, China). Animals were fed in an environment with a pressure ventilation system, an ambient temperature maintained at 22 ± 2 °C, and a relative humidity maintained at 40%–70%. Mice were housed in a 12-h/12-h light/dark cycle (8:00–20:00) and provided with standard mouse chow and water ad libitum. The behavioral tests were mainly carried out in the experimental animal operating room from 9:00 a.m. to 16:00 p.m., and the time points were adjusted according to the experimental designs. All animal experiments were conducted according to the National Institutes of Health Laboratory Animal Care and Use Guidelines. All animal operations and experimental procedures were approved by the Animal Ethics Committee of Soochow University.

### Drugs and administration

Botulinum toxin type A was provided by Lanzhou Institute of Biological Products Co., Ltd. Reserpine injections were purchased from Tianjin, Jin Yao Pharmaceutical Co., Ltd. The mice were fed a reserpine solution (prepared with drinking water, 3 μg/mL) daily for 10 weeks. All mice were treated with BoNT/A (10 U/kg), which was injected into the cheek once per day for three consecutive days at the 10^th^ week.

Pramipexole and duloxetine were obtained from Boehringer Ingelheim Pharma GmbH & Co. KG and Lilly Del Caribe. Inc, respectively. Mice received intraperitoneal injections of pramipexole and duloxetine (10 mg/kg) for two consecutive weeks starting at the 10^th^ week of reserpine treatment.

### Behavioral tests

#### Body weight

Body weight was recorded with an electronic balance at the indicated times.

#### Rotarod test

A rotarod apparatus (XR-6C, Shanghai Xinruan Information Technology Co., Ltd., Shanghai, China) was used to measure motor ability. During the training period, each mouse was trained for 5 min on an accelerating rotarod that started at a low speed for acclimation and then accelerated at a constant rate of 20 rpm until the mouse fell from the rod. Mice underwent one trial per day for three consecutive days. After all the mice were trained, they were tested in three trials at 20 rpm with a 15-min rest between each trial. The latency to fall was recorded for each trial, and the average of three trials was analyzed.

#### Pole climbing test

The apparatus for the pole test was a 50-cm-high and 0.5-cm-diameter wooden pole, wrapped with gauze to avoid slipping and placed on the bench. The mice were positioned on the top of the wooden pole and allowed to climb down freely. The time of climbing down from the top of the pole was recorded. Each animal was tested for three successive trials with a 10-min intertrial interval. The average time of the three trials was calculated for statistical analyses.

#### Open field test

An acrylic box (40 cm × 40 cm × 40 cm, L × W × H) of the open field test apparatus was used to evaluate locomotor activity. Each mouse was placed in the same corner of the box and allowed to explore the environment for 10 min. The arena was cleaned with 75% EtOH between each trial. The data were automatically recorded and analyzed by video tracking software (XR-XZ301, Shanghai XinRuan Information Technology Co., Ltd., China) to measure exploratory behavior and locomotor activity.

#### Forced swimming test

The forced swimming test (FST) was performed as previously described [31]. Briefly, mice were individually kept in a 2-L beaker filled with 1.5 L water at 24–26 °C and allowed to swim for 6 min. Immobility time was defined as the absence of movement except for the necessary procedure to keep the mouse afloat and recorded during the last 4 min. Finally, mice were placed in their home cages after careful drying with a hairdryer.

#### Tail suspension test

The tail suspension test (TST) was performed according to a previously described method [31]. Briefly, mice were suspended by the tail with adhesive tape at 50 cm above the floor for 6 min. After 1 min of acclimation, the behavioral test was conducted, and the immobility time was recorded during the last 4 min.

#### Sucrose preference test

The sucrose preference test is widely used to detect anhedonia in mice and was conducted as previously reported [31]. Mice were housed individually and habituated to drinking water from two bottles. After 24 h, one of the drinking water bottles was exchanged with a bottle filled with 1% sucrose. The location of the two bottles was switched 24 h later. After the training phase, mice were deprived of water and food for 24 h. The bottles filled with drinking water or 1% sucrose were weighed and placed randomly before the test, and their positions were exchanged at 12 h. After 24 h, the bottles were weighed again to measure the liquid consumed from each bottle. The sucrose preference ratio was calculated by the following equation: sucrose preference ratio (%) = sucrose intake/(sucrose intake + water intake) × 100%.

### Cell culture

The mouse BV2 microglial cell line was obtained from the Institute of Neuroscience at Soochow University and cultured in high-glucose DMEM (SH30243.01, HyClone, USA) containing 10% FBS (16000-044, Gibco, USA) in a humidified atmosphere under 5% CO_2_ at 37 °C. Reserpine was prepared at a concentration of 50 nM using 0.9% saline containing 10% DMSO. BoNT/A was dissolved in 0.9% saline and prepared in three concentrations of 0.01 U/mL, 0.1 U/mL, and 0.5 U/mL. Cell culture media was supplemented with reserpine in the presence or absence of BoNT/A for 24 h.

### CCK-8 assay

BV2 cells were seeded at a density of 5000/well in a 96-well plate for the CCK-8 assay. Ten microliters of BoNT/A (0.01 U/mL, 0.1 U/mL, and 0.5 U/mL) was added to the BV2 cell culture separately in triplicate wells, and BV2 cells were incubated for 24 h. Ten microliters of CCK-8 solution (NCM Biotech, China) was finally added to each well. Cell incubation continued for another 4 h. Cell viability was calculated by the absorbance at 450 nm.

### High-performance liquid chromatography (HPLC) analysis of dopamine levels

The dopamine level in the substantia nigra pars compacta (SNpc) was determined by HPLC. Briefly, the brain was dissected and homogenized in 400 mL of 0.4 mol/L perchloric acid with an ultrasonic homogenizer (Microsonic, Dortmund, Germany). Afterward, the homogenates were centrifuged at 15,000 rpm for 20 min at 4 °C. The obtained supernatant was then filtered through a 0.22 μm syringe filter to be stored at 80 °C for further chemical analysis. The concentrations of DA were determined by a reverse-phase HPLC equipped with a C18 column (TC-C18, Agilent, Middelburg, Netherlands) in a mobile phase composed of 0.1 mol/L NaAc containing 0.1 mol/L EDTA-Na2 and 10% methanol at pH 5.1. The flow rate was set at 1 mL/min. The data were analyzed by the area under the peak and referenced to that of an external standard. The results are shown in nanograms per gram of wet tissue (ng/g).

### Enzyme-linked immunosorbent assay (ELISA)

The substantia nigra pars compacta and hippocampus were homogenized in lysis buffer. The homogenate or BV2 cell media was centrifuged at 4000 rpm for 20 min. The dopamine (DA) and proinflammatory cytokine (TNF-α and IL-1β) levels were quantified by ELISA kits. The mouse DA ELISA kit (ZC-38119), mouse C1q ELISA kit (LV30667L), mouse TNF-α ELISA kit (LV30536L), and mouse IL-1β ELISA kit (LV30300L) were obtained from ZCIBIO Technology and Shanghai DCELL Biologics separately. The experimental procedure was performed according to the manufacturer’s instructions.

### Western blotting

The individual substantia nigra pars compacta and hippocampus were rapidly dissected and homogenized in lysis buffer containing phosphatase inhibitors and a cocktail of protease inhibitors. The total protein concentrations were assayed by the BCA protein assay kit (Pierce, Rockford, IL). The protein extract was loaded on 10% polyacrylamide gels and electrophoresed at 80 V for 30 min and 120 V for 2 h, separately. Proteins were transblotted onto polyvinylidene difluoride membranes and blocked in 5% nonfat milk in TBST. Membranes were incubated overnight at 4 °C with the following primary antibodies: rabbit anti-tyrosine hydroxylase (Cell Signaling Technology, 58844 S, 1:1000), rabbit anti-C1q (Abcam, ab182451, 1:1000), mouse anti-C3 (Protech, 66157-1-AP, 1:1000), mouse anti-tubulin (Abcam, ab7291, 1:1000), and mouse anti-GAPDH (Mesgen Biotechnology, MAN1002, 1:1000). Membranes were incubated for 1 h at room temperature with the following secondary antibodies: goat anti-rabbit IgG (H + L) HRP (Mesgen, MAN4001, 1:2500) and goat anti-mouse IgG (H + L) HRP (Mesgen, MAN4002, 1:2500). Protein bands were visualized using an enhanced chemiluminescence detection kit (Thermo Fisher Scientific, 32106). The intensity of each band was analyzed using Tanon 5200 Multi (Tanon, Shanghai).

### Quantitative real-time RT‒PCR

Total RNA was extracted by the TRIzol reagent (Invitrogen, 15596026) according to the manufacturer’s protocol. Total RNA was reverse transcribed to synthesize cDNA using a RevertAid First Strand cDNA Synthesis Kit (Thermo Fisher Scientific, K1622). qRT‒PCR was performed with 2x SYBR Green qPCR Master Mix (Bimake, B21703). Reactions were performed in triplicate and analyzed with an ABI 7500 Real-Time PCR system. The primer sequences for corresponding genes were as follows: IL-1β (FW, CTTCAGGCAGGCAGTATCACTCAT; RV, TCTAATGGGAACGTCACACACCAG), TNF-α (FW, CGGGCAGGTCTACTTTGGAG; RV, ACCCTGAGCCATAATCCCCT), C3 (FW,TTCTCCGCAGAGTTTGAGGT; RV, TTCTTATCGCCATCCTGGAC), C1qa (FW, CTGGCATCCGGACTGGTATC; RV, CTTTCACGCCCTTCAGTCCT), C3aR (FW, CCCCAAGACATTGCCTCCAT; RV, GACTGTGTTCACGGTCGTCT), GAPDH (FW, TGTGAACGGATTTGGCCGTA; RV, GGCCTCACCCCATTTGATGT), CX3CR1 (FW, AGAGCCGTCAGACTCATCCT; RV, CCCGGCAAAGGCGTAGATAA); and CX3CL1 (FW, ACGAAATGCGAAATCATGTGC; RV, CTGTGTCGTCTCCAGGACAA). All mRNA levels of these genes were normalized to the expression of endogenous GAPDH and calculated by the 2 (-ΔΔCT) method.

### Immunohistochemistry

Immunohistochemistry analyses were performed as previously described [36]. Briefly, mice were anesthetized with Avertin (0.5 mg per g) and transcardially perfused with PBS and 4% paraformaldehyde (PFA) in PBS. Brains were postfixed in 4% PFA overnight and cryoprotected in a 30% PBS-buffered sucrose solution. Coronal sections (30 μm thickness) were sliced using a sliding-freezing microtome (SM 2010R, Leica, Germany). Sections were first rinsed in PBS, washed in 0.025% Triton X-100 in PBS (PBST) and then blocked in 5% normal goat serum in PBST followed by incubation with primary antibodies. Sections were further washed with PBST and incubated with secondary antibodies. After washing in PBST and PBS, sections were mounted and imaged by an LSM 800 laser-scanning confocal microscope (Carl Zeiss, Germany). The primary antibodies were as follows: rabbit anti-TH (Cell Signaling Technology, 58844 S, 1:1000), rabbit anti-C1q (Abcam, ab182451, 1:1000), mouse anti-C3 (Protech, 66157-1-AP, 1:1000), guinea pig anti-VGlut2 (Synaptic Systems, 135-404, 1:1000), mouse anti-PSD95 (Synaptic Systems, 124-011, 1:1000), guinea pig anti-VGAT (Synaptic Systems, 131-004, 1:1000), mouse anti-Gephyrin (Synaptic Systems, 147-021, 1:1000), rabbit anti-Iba1 (Wako Chemicals, 019-19741, 1:1000), and rat anti-CD68 (Abcam, ab53444, 1:1000). The following secondary antibodies were used: goat anti-rabbit IgG Alexa-488 (Abcam, ab150080, 1:500), goat anti-rat IgG Alexa-647 (Cell Signaling Technology, 4418, 1:500), goat anti-mouse IgG1 FITC (Abcam, ab97239, 1:500), and goat anti-guinea pig IgG Alexa 555 (Thermo Fisher Scientific, A-21435, 1:500). All sections were stained with DAPI (Thermo Fisher Scientific, D1306, 1:500). ImageJ (NIH) was used for image processing. For C1q and C3 deposits, the intensities of individual C1q and C3 puncta were quantified. For synaptic density, colocalized images were generated by the Process “Image Calculator” tool to multiply the post-threshold presynaptic and postsynaptic images, and the intensity of the colocalized image was then quantified.

### Microglial activation and engulfment of synapses

Immunostaining of the presynaptic marker VGlut2, lysosomal marker CD68, and microglial marker Iba1 was performed as described above. For microglial activation, CD68 occupancy within microglia was quantified by Imaris Reconstruction software (V.9.9.1, Bitplane) using a previously described method [36]. The percentage of CD68 occupancy within microglia was calculated by the following formula: volume of surface-rendered CD68/volume of surface-rendered Iba1^+^ cells. For the VGlut2 engulfment assays, the percentage of VGlut2 phagocytosed by microglia was quantified by the following formula: volume of surface-rendered engulfed synapses in lysosomes/volume of surface-rendered Iba1^+^ cells. Microglial morphology was characterized and analyzed using the “analyze skeleton” plug-in function in FIJI [37, 38]. Briefly, Iba1^+^ cells in brain tissue sections were observed using a confocal microscope with z-stack acquisition and a 63× objective. Only the microglia that had clear somas and processes were utilized for statistical analysis. All the photomicrographs of interest were converted into 8-bit images and processed into binary and skeletonized images. A cutoff value was defined at five pixels so that microglia with a length of branches less than the set value were excluded from the analysis. The number of branches of microglial processes and their length were quantified. Microglial characteristics were estimated from two to three sections per animal for statistical comparisons. Representative images were captured in sections and are presented. Microglial soma was traced by Imaris reconstruction software (V.9.9.1, Bitplane) using the previously described method [36], and soma volumes were measured based on the surface-rendered images.

### Golgi staining

An FD Rapid GolgiStain™ Kit (PK401) was used in this study. The experimental procedure was conducted according to the manufacturer’s instructions. Briefly, brains were immediately dissected and washed in Milli-Q water to remove blood from the surface. The brains were rinsed in solution A and B mixture (1:1) for 10 days and immersed in solution C. Brains were sectioned at 100 μm using a sliding-freezing microtome (SM 2010R, Leica, Germany) and mounted on gelatin-coated slides. Golgi-stained sections were imaged under bright-field illumination using a Zeiss confocal microscope LSM880. The spine density (the number of spines per dendrite length in 10 μm) of pyramidal neurons located in the CA1 region of the hippocampus was determined.

### Statistical analysis

GraphPad Prism V.8 was applied for all statistical analyses. All data are presented as the mean ± SEM. Data from pramipexole and reserpine treatment in the FST analysis (supplementary data) are the same batch analyzed in our recent study [39]. Differences between two groups were determined with an unpaired two-tailed Student’s *t* test. When comparing more than two groups, one-way ANOVA was performed by Tukey’s post hoc test comparing every mean with every other mean. Two-way ANOVA was used by the Bonferroni post hoc test comparing all means to the control.

## Results

### Reserpine induced depression-like behavior in a mouse model of Parkinson’s disease

**Fig. 1:**
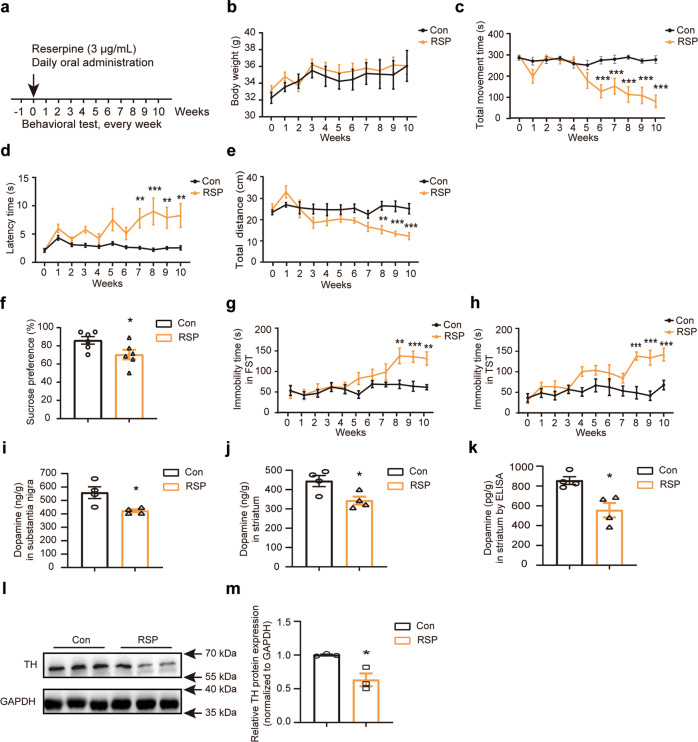
Reserpine-induced behavioral outcomes and neurochemical features of depression in a mouse model of Parkinson’s disease. **a** Experimental paradigm for behavioral testing. **b** The bodyweight of mice (*F*_interaction (10, 198)_ = 0.1216, *P* = 0.9995). **c** The total movement time in the rotarod test (*F*_interaction (10, 176)_ = 6.1666, *P* < 0.0001). **d** The latency time in the pole climbing test (*F*_interaction (10, 176)_ = 2.281, *P* = 0.0155). **e** The total distance traveled in the open field test (*F*_interaction (10, 154)_ = 3.442, *P* = 0.0004). **f** Performance in the SPT (*t* = 2.333, df = 10, *P* = 0.0419). **g** Performance in the FST (*F*_interaction (10, 154)_ = 2.805, *P* = 0.0032). **h** Performance in the TST (*F*_interaction (10, 154)_ = 3.306, *P* = 0.0007). **i**, **j** Substantia nigra (**i**) and striatal (**j**) dopamine concentrations were detected by HPLC (for substantia nigra dopamine, *t* = 3.005, df = 6, *P* = 0.0239. For striatal dopamine, *t* = 2.884, df = 6, *P* = 0.0279). **k** Striatal dopamine concentrations were determined by ELISA (*t* = 3.614, df = 6, *P* = 0.0112). **l** Immunoblotting analysis of the expression of TH in the SNpc. **m** Quantification of TH protein expression normalized to GAPDH in the SNpc (*t* = 3.813, df = 4, *P* = 0.0189). All data are presented as the mean ± SEM. *n* = 6–10 mice for each group in the behavioral test. *n* = 3–6 mice for each group in the neurochemical test. Two-way ANOVA followed by Bonferroni’s multiple comparison test and an unpaired Student’s *t* test were used. *P  <  0.05, **P  <  0.01, ***P  <  0.001, versus Con group.

The reserpine-induced model has been widely applied to investigate the role of the monoamine system in the regulation of motor and psychiatric disorders, including PD and depression [40, 41]. Thus, we used chronic administration of reserpine to model progressive depressive symptoms in Parkinson’s disease. To investigate the effects of chronic reserpine administration on various behavioral outcomes, we treated mice with reserpine in drinking water for 10 weeks (Fig. 1a). All mice were weighed on the day before the behavioral tests. The reserpine treatment did not significantly change the body weight of the mice (Fig. 1b). We used multiple cohorts of mice for behavioral tests, including the rotarod test, pole climbing test, and open field test, to evaluate the effect of reserpine on locomotion. Compared to the control group, the total time spent on the rotating rod significantly decreased after the 6^th^ week in the reserpine-treated mice (Fig. 1c). Changes in bradykinesia were measured by the pole climbing test. The latency time was shown to be significantly increased after the 7^th^ week in the reserpine group (RSP) compared to the control group (Con) (Fig. 1d). The locomotor activity of the mice was determined by the open field test. The total traveled distance significantly declined after the 8^th^ week in the reserpine-treated mice (Fig. 1e).

To further explore the depressive-like phenotype induced by reserpine treatment, we used the sucrose preference test (SPT), forced swimming test (FST), and tail suspension test (TST). Reserpine-treated mice showed a significantly reduced percentage of sucrose consumption compared to control mice (Fig. 1f). In addition, compared with the control mice, the reserpine-treated mice also exhibited a longer immobility time after the 8^th^ week in the FST and TST (Fig. 1g, h). These results indicated that chronic administration of reserpine could reproduce remarkable depression-like symptoms.

To investigate whether reserpine treatment impacts the secretion of dopamine in the substantia nigra and striatum, the dopamine level was detected by HPLC. The dopamine level was significantly reduced in both the substantia nigra (Fig. 1i) and striatum (Fig. 1j). Moreover, the change in dopamine level in the striatum was confirmed with a significant reduction by ELISA (Fig. 1k). We also measured the levels of tyrosine hydroxylase (TH) in the SN. The protein level of TH was significantly decreased in the reserpine-treated mice (Fig. 1l, m). Taken together, these behavioral and neurochemical assays findings suggested that the chronic administration of reserpine could produce depressive-like behavior and pathological changes in the mouse model of Parkinson’s disease.

### BoNT/A ameliorated depressive-like behavior but not TH activity in the reserpine-induced mouse model of Parkinson’s disease

**Fig. 2:**
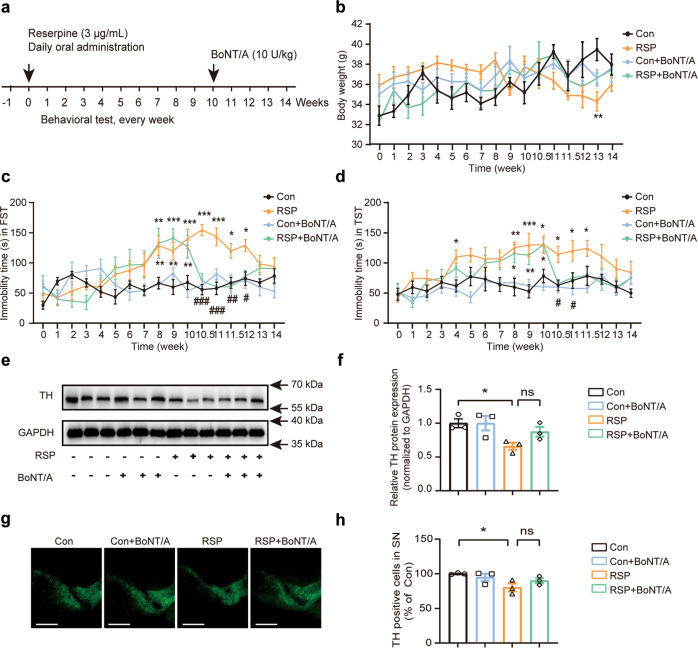
BoNT/A treatment ameliorated reserpine-induced depressive-like behavior but not TH activity in mice. **a** Experimental paradigm for behavioral testing. Reserpine was prepared in drinking water at 3 μg/mL and administered to male ICR mice (6–8 weeks old). The depressive-like behavioral test was performed every week for 10 weeks. BoNT/A was injected into the mouse cheek (10 U/kg) once per day for three consecutive days. Depressive-like behavior was tested three days later and every week until the 14th week. **b** The effect of BoNT/A treatment on body weight was measured (*n* = 8 mice for each group, *F*_interaction (48, 476)_ = 1.879, *P* = 0.0006). **c**, **d** Effect of BoNT/A treatment on performance in the FST (**c**) and TST (**d**) (*n* = 8 mice for each group, FST, *F*_interaction (48, 476)_ = 3.320, *P* < 0.0001. For TST, *F*_interaction (48, 476)_ = 1.487, *P* = 0.0222). **e** Immunoblotting analysis of the expression of TH in the substantia nigra pars compacta (SNpc) with BoNT/A treatment in reserpine-treated mice. **f** Quantification of TH protein expression normalized to GAPDH in the SNpc (*n* = 3 mice for each group, *F*_(3, 8)_ = 4.316, *P* = 0.0436). **g** Representative images of tyrosine hydroxylase (TH)-stained neurons in the SNpc. Scale bar = 100 μm. **h** Quantification of TH-positive neurons relative to the Con group in the SNpc (*n* = 3 mice for each group, *F*_(3, 8)_ = 3.767, *P* = 0.0593). All data are presented as the mean ± SEM. Two-way ANOVA followed by Bonferroni’s multiple comparison test and one-way ANOVA with Tukey’s post hoc test were used. *P < 0.05, **P < 0.01, ***P < 0.001, versus Con group. ^#^*P* < 0.05, ^##^*P* < 0.01, ^###^*P* < 0.001.

The safety and efficacy of BoNT/A have been evaluated in clinical trials for the treatment of depression [29], and we previously demonstrated that a single facial injection of BoNT/A could induce a rapid and prolonged improvement in depression-like behaviors in a chronic mild-stress mouse model [31]. To determine the effect of BoNT/A on depressive-like behavior in Parkinson’s disease, mice were continuously administered reserpine (3 μg/mL) in water for 10 weeks and infused with BoNT/A once at the 10^th^ week. After three consecutive days of BoNT/A injections, the effect of BoNT/A on depressive-like behaviors in the mice was evaluated by the FST and TST (Fig. 2a). No significant difference in the bodyweight of the mice was observed in the first 12 weeks (Fig. 2b). The differences in the immobility time in the FST and TST were observed from the 8^th^ week and maintained until the 12th week after reserpine administration was withdrawn at the 10^th^ week (Fig. 2c, d). BoNT/A treatment (RSP + BoNT/A) significantly reduced the immobility time of mice in the FST and TST compared to RSP alone (Fig. 2c, d). This result indicated that the depressive-like behavior of BoNT/A-treated mice recovered much faster than that of the untreated mice. In addition, the effect of BoNT/A on reserpine-induced depressive symptoms in PD mice was compared with that of other common medications, including pramipexole and duloxetine. In the forced swimming test, both pramipexole and duloxetine treatment significantly reduced the immobility time in the reserpine-treated mice. Furthermore, the effect of BoNT/A on the immobility time in the FST was comparable to that of pramipexole and duloxetine (Fig. S1).

To investigate whether BoNT/A treatment impacts neurochemical features in Parkinson’s disease, the levels of TH were evaluated by Western blotting and immunohistochemistry. A significant reduction in TH protein levels in the substantia nigra was observed in the RSP group compared with the control group. The decreased TH protein levels in the RSP group were reversed after BoNT/A treatment, but the difference was not significant (Fig. 2e, f). The decreased TH-positive cells in the substantia nigra in the reserpine-treated group were also partially rescued with BoNT/A treatment (Fig. 2g, h). Therefore, our results revealed that BoNT/A treatment significantly ameliorated depressive-like behavior but did not markedly improve TH activity in the mouse model of depression in Parkinson’s disease.

### BoNT/A ameliorated the reserpine-induced activation of the classical complement pathway

**Fig. 3:**
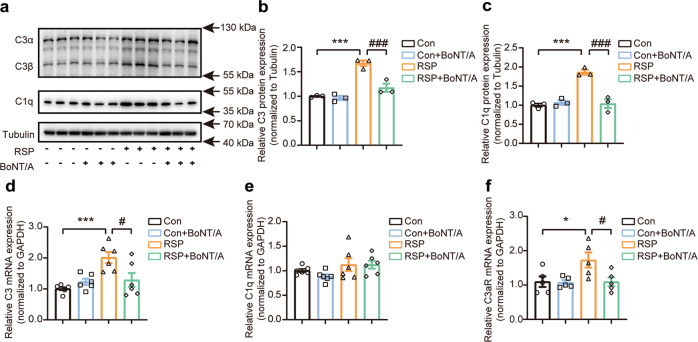
BoNT/A treatment inhibited complement expression in the reserpine-treated mouse hippocampus. **a** Immunoblotting analysis of the expression of complement proteins C3 and C1q. **b**, **c** Quantification of complement C3 protein (**b**) and C1q protein (**c**) expression normalized to Tubulin (*n* = 3 mice for each group, For C3, *F*_(3, 8)_ = 34.09, *P* < 0.0001. For C1q, *F*_(3, 8)_ = 29.37, *P* = 0.0001). **d**-**f** Complement C3 (**d**), C1q (**e**), and C3aR (**f**) mRNA expression levels were determined by qRT‒PCR (*n* = 5–6 mice for each group, For C3, *F*_(3, 20)_ = 8.045, *P* = 0.0010. For C1q, *F*_(3, 20)_ = 1.978, *P* = 0.1498. For C3aR, *F*_(3, 16)_ = 4.338, *P* = 0.0204). All data are presented as the mean ± SEM. One-way ANOVA with Tukey’s post hoc test was used. *P  <  0.05, ***P  <  0.001, versus Con group. ^#^*P*  <  0.05, ^###^*P*  <  0.001, versus the RSP group.

Recent studies from clinical and animal models have identified a significant increase in complement C3 expression in the prefrontal cortex of depressed suicidal individuals and a stress-induced mouse model of depression [25]. To better understand the correlation between complement-microglia-mediated synapse loss and hippocampal atrophy in depression in Parkinson’s disease [42], we first investigated the expression of complement proteins in the classical complement pathway in the reserpine-treated mice. In our studies, we found a striking increase in complement C3 and C1q protein expression in the hippocampus of reserpine-treated mice compared to control mice. BoNT/A treatment significantly decreased complement C3 and C1q protein expression in the RSP group (Fig. 3a–c). The mRNA expression of complement C3 was found to have a consistent change compared to that of protein alterations (Fig. 3d). In contrast, no significant difference was found in the complement C1q mRNA expression levels among the different groups (Fig. 3e). The complement receptor C3aR, a signaling receptor expressed by microglia, can be activated by the C3 cleavage fragment C3a and has been reported to have important roles in chronic unpredictable mild stress (CUMS) mice [43–46]. We found that the mRNA expression of C3aR was significantly increased in the RSP group but declined in the RSP group treated with BoNT/A (Fig. 3f).

**Fig. 4:**
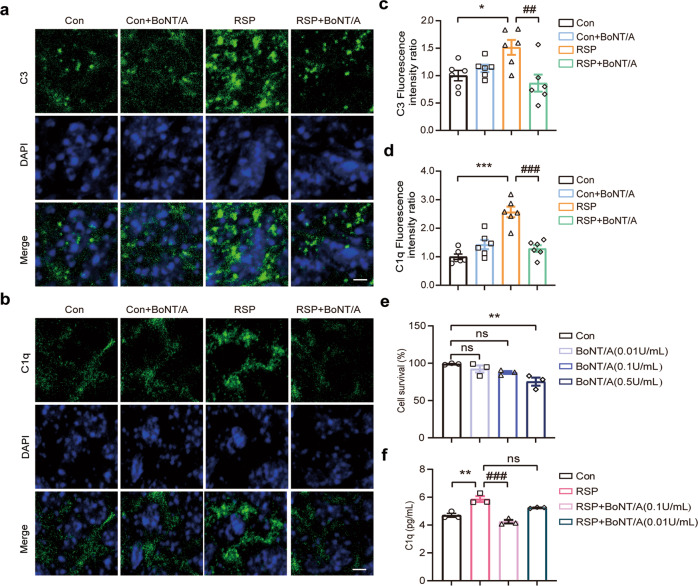
BoNT/A treatment inhibited the activation of the classical complement pathway in vivo and in vitro. **a**, **b** Immunostaining of complement C3 (**a**) and C1q (**b**) in the hippocampal CA1 regions. Scale bar = 5 μm. **c**, **d** Quantification of complement C3 (**c**) and C1q (**d**) fluorescence intensity (*n* = 6 images from 3 mice for each group. For C3, *F*_(3, 20)_ = 5.604, *P* = 0.0059). For C1q, *F*_(3, 20)_ = 22.60, (*P* < 0.0001). **e** Cell viability of BV2 microglial cells exposed to BoNT/A (0.01 U/mL, 0.1 U/mL, and 0.5 U/mL) for 24 h was determined by the CCK-8 assay (*n* = 3 replicates for each group. *F*_(3, 8)_ = 7.122, *P* = 0.0120). **f** Complement C1q in the cell media secreted by BV2 microglial cells was detected by ELISA (*n* = 3 replicates for each group. *F*_(3, 8)_ = 23.71, *P* = 0.0002). All data are presented as the mean ± SEM. One-way ANOVA with Tukey’s post hoc test was used. *P  <  0.05, **P  <  0.01, ***P  <  0.001, versus Con group. ^##^*P*  <  0.01, ^###^*P*  <  0.001, versus RSP group.

Next, we examined the effect of BoNT/A on the activation of the classical complement pathway. The results from the immunohistochemistry analysis of the hippocampal CA1 region showed dramatically increased deposits of complement C3 and C1q in the reserpine-treated mice. In contrast, BoNT/A treatment significantly alleviated the elevated complement C3 and C1q deposits in the reserpine-treated mice (Fig. 4a–d). Moreover, microglia are recognized as the primary source of complement C1q. Therefore, we estimated the effect of BoNT/A on the secretion of complement C1q induced by reserpine in BV2 cell media using ELISA. We first evaluated the cell survival rate of BV2 cells treated with different doses of BoNT/A by CCK-8 assay, and toxic effects on the cell survival rate were found at a dose of 0.5 U/mL but not 0.01 U/mL or 0.1 U/mL (Fig. 4e). Thus, we employed the two doses of 0.01 U/mL and 0.1 U/mL for further studies. The ELISA results showed that reserpine significantly stimulated the secreted levels of complement C1q. In contrast, the elevated complement C1q significantly declined with BoNT/A treatment at 0.1 U/mL (Fig. 4f). These results suggest that BoNT/A can ameliorate reserpine-induced complement activation in vivo and in vitro.

### BoNT/A alleviated reserpine-induced microglial activation

**Fig. 5:**
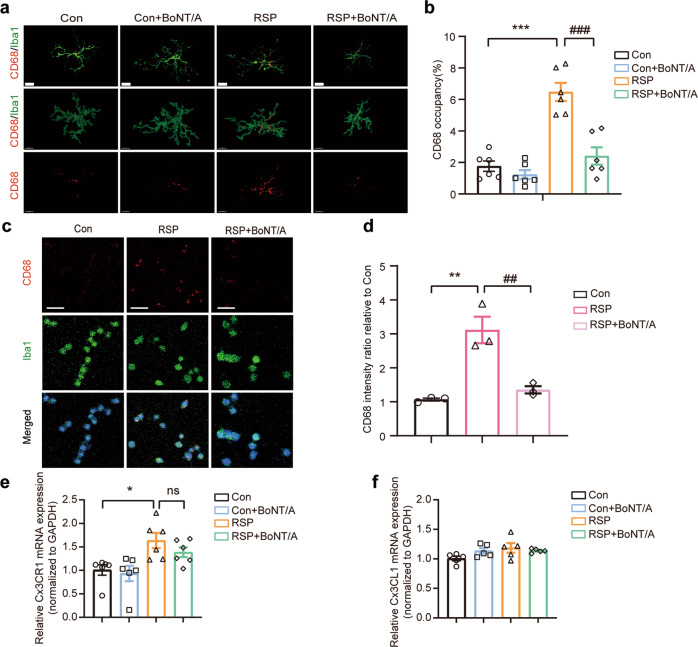
BoNT/A treatment suppressed microglial activation in vivo and in vitro. **a** Representative immunostaining images of the microglial marker Iba1 (green) and lysosomal marker CD68 (red) in the hippocampal CA1 regions (top) and corresponding 3D reconstructed images. Scale bar = 10 μm. **b** Quantification of the percentage of CD68^+^ lysosome volume in Iba1^+^ microglia volume (*n* = 6 images from 3 mice for each group. *F*_(3, 20)_ = 27.82, *P* < 0.0001). **c** Representative immunostaining images of the microglial marker Iba1 (green) and lysosomal marker CD68 (red) in BV2 microglial cells exposed to 50 nM reserpine (RSP) for 24 h in the presence or absence of BoNT/A (0.1 U/mL). Scale bar = 50 μm. **d** Quantification of the relative CD68^+^ lysosome fluorescence intensity in Iba1^+^ microglia (*n* = 3 replicates for each group). *F*_(2, 6)_ = 22.26, (*P* = 0.0017). **e**, **f** Chemokine receptor CX3CR1 (**e**) and fractalkine CX3CL1 (**f**) mRNA expression levels in the hippocampal samples were measured by qRT‒PCR (*n* = 5–6 mice for each group. For CX3CR1, *F*_(3, 20)_ = 5.718, *P* = 0.0054). For CX3CL1, *F*_(3, 16)_ = 2.236, (*P* = 0.1235). All data are presented as the mean ± SEM. One-way ANOVA with Tukey’s post hoc test was used. *P  <  0.05, **P  <  0.01, ***P < 0.001, versus Con group. ^##^*P*  <  0.01, ^###^*P*  <  0.001, versus RSP group.

To further investigate the effect of complement activation on microglia, we performed immunohistochemistry analyses of microglia. Ionized calcium binding adaptor molecule 1 (Iba1) is a widely used protein marker of both resting and activated microglia. CD68 is a lysosomal-associated protein in microglia in the brain and is associated with phagocytic cells. Thus, we used both Iba1 and CD68 to define microglial activation. Our staining results showed that microglial activation was most pronounced in the hippocampus of reserpine-treated mice compared to control mice (Fig. 5a, b). In contrast, BoNT/A treatment significantly reduced the lysosomal volume in microglia (Fig. 5a, b). Next, we performed microglial morphological characterization using semiautomated software analysis of the number of branching points and length of microglial processes and cell soma volume. As shown in the representative images (Fig. S2a), 3-dimensional surface rendering and skeletonization of individual microglia were utilized to evaluate the cell soma volume, number of branching points, and branch length of microglia. Overall, an increase in cell soma size and a reduction in the number of branching points were found in the hippocampus of reserpine-treated mice, which were reversed after BoNT/A treatment (Fig. S2b, c). No difference in the length of microglial processes was observed after BoNT/A treatment (Fig. S2d).

Moreover, to confirm the effect of BoNT/A on microglial activation, we also evaluated the lysosomal intensity in BV2 microglial cells in vitro. The in vitro results showed a distinct increase in CD68 intensity in microglia after stimulation with reserpine (Fig. 5c, d). Subsequent treatment with BoNT/A significantly suppressed reserpine-induced BV2 microglial cell activation (Fig. 5c, d). These results indicated that BoNT/A could significantly inhibit reserpine-induced microglial activation in vivo and in vitro. The chemokine receptor CX3CR1 is selectively expressed in microglia, and the CX3CL1/CX3CR1 signaling pathway is involved in microglial activation [47]. As we observed microglial activation induced by reserpine in the hippocampus, we determined the alteration of CX3CL1/CX3CR1 signaling. We found that the mRNA expression of CX3CR1 was significantly increased in the RSP group but reduced after BoNT/A treatment (Fig. 5e). However, no remarkable changes were observed in the mRNA expression of CX3CL1 among the different groups (Fig. 5f). Taken together, our results indicate that BoNT/A can alleviate reserpine-induced microglial activation in vivo and in vitro.

### BoNT/A ameliorated synapse loss in reserpine-treated mice

**Fig. 6:**
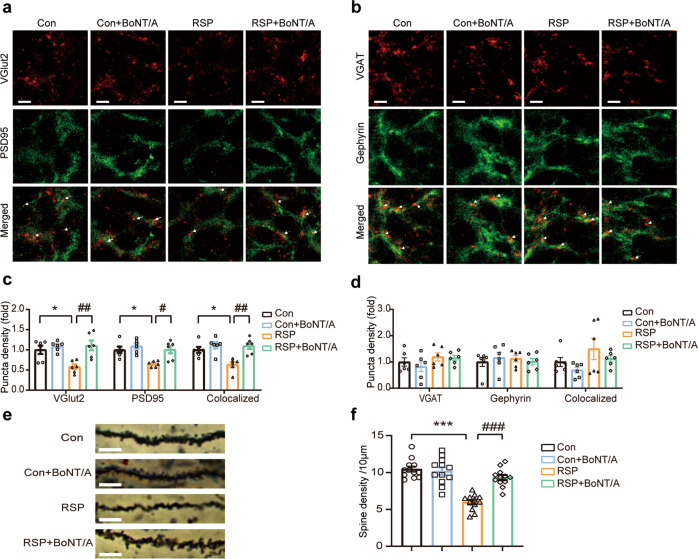
BoNT/A treatment alleviated synapse and spine loss in the reserpine-treated mouse hippocampus. **a**, **b** Representative immunostaining images of excitatory presynaptic marker VGlut2 (red) and postsynaptic marker PSD95 (green) (**a**) and inhibitory presynaptic marker VGAT (red) and postsynaptic marker Gephyrin (green) (**b**) in the hippocampal CA1 regions. Scale bar = 5 μm. **c**, **d** Quantification of individual and colocalized excitatory pre- and postsynaptic markers (**c**) and inhibitory pre- and postsynaptic markers (**d**) (*n* = 6 images from 3 mice for each group. For VGlut2, *F*_(3, 20)_ = 7.739, *P* = 0.0013. For PSD95, *F*_(3, 20)_ = 7.603, *P* = 0.0014. For VGlut2 + PSD95, *F*_(3, 20)_ = 9.593, *P* = 0.0004. For VGAT, *F*_(3, 20)_ = 1.191, *P* = 0.3384. For Gephyrin, *F*_(3, 20)_ = 0.2680, *P* = 0.8476. For VGAT + Gephyrin, *F*_(3, 20)_ = 2.016, *P* = 0.1440). **e** Representative images of Golgi-stained dendrite spines of pyramidal neurons in the hippocampal CA1 regions. Scale bar = 5 μm. **f** Quantification of the dendrite spine density of pyramidal neurons in the hippocampal CA1 regions (*n* = 12 images from 3 mice for each group. *F*_(3, 44)_ = 27.26, *P* < 0.0001). All data are presented as the mean ± SEM. One-way ANOVA with Tukey’s post hoc test was employed. *P  <  0.05, ***P  <  0.001, versus Con group. ^#^*P*  <  0.05, ^##^*P*  <  0.01, ^###^*P*  <  0.001, versus the RSP group.

Emerging evidence has indicated that depression symptoms in Parkinson’s disease are associated with hippocampal atrophy [42]. It is thought that hippocampal atrophy begins with CA1 synapse loss [48]. VGlut2 and VGAT are excitatory and inhibitory presynaptic vesicle proteins, respectively. PSD95 primarily regulates the differentiation of excitatory synapses at the postsynaptic density, and Gephyrin is a central GABAergic synapse organizer. To test whether synapse loss occurs in the hippocampal CA1 region in reserpine-induced depression in a Parkinson’s disease mouse model, we measured the synaptic density of colocalized puncta of VGlut2 and PSD95 at excitatory synapses and colocalized puncta of VGAT and Gephyrin at inhibitory synapses (Fig. 6a, b). The colocalized puncta of VGlut2 and PSD95 were significantly reduced in the reserpine-treated hippocampus. In contrast, BoNT/A treatment significantly reversed the reduction in colocalized puncta in the RSP group (Fig. 6c). However, no significant changes in the colocalized puncta of VGAT and Gephyrin were observed (Fig. 6d). To further understand whether this loss of synaptic density was associated with a loss of spines, we quantified spine numbers on apical dendrites of Golgi-impregnated pyramidal CA1 neurons (Fig. 6e). We found a dramatically decreased density of dendritic spines from CA1 pyramidal neurons in the reserpine-treated group, while spine loss was significantly rescued after the infusion of BoNT/A (Fig. 6f). These results indicate that synapse and spine loss emerged in the hippocampal CA1 region in reserpine-treated mice and could be reversed with BoNT/A treatment.

**Fig. 7:**
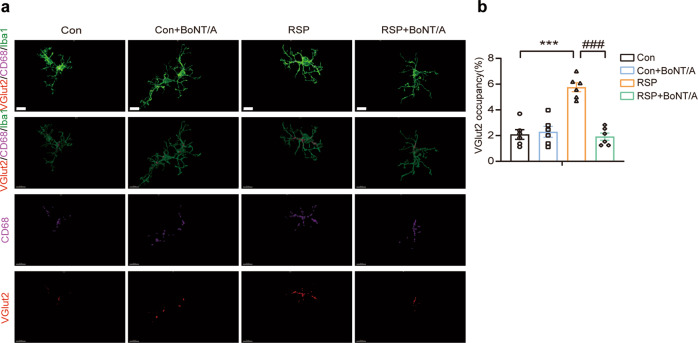
BoNT/A treatment alleviated synapse and spine loss in the reserpine-treated mouse hippocampus. **a** Representative immunostaining images of presynaptic marker VGlut2 (red), lysosomal marker CD68 (magenta), and microglial marker Iba1 (green) in the hippocampal CA1 regions, the corresponding 3D construction of VGlut2 (red), CD68 (magenta), and Iba1 (green) channels by Imaris, as well as 3D reconstructed images of the microglial lysosome contents (magenta) and engulfed synaptic contents (red). Scale bar = 10 μm. **b** Quantification of the percentage of engulfed VGlut2^+^ synaptic volume in Iba1^+^ microglial volume (*n* = 6 images from 3 mice for each group. *F*_(3, 20)_ = 25.95, *P* < 0.0001). All data are presented as the mean ± SEM. One-way ANOVA with Tukey’s post hoc test was applied. ***P  <  0.001, versus Con group. ^###^*P*  <  0.001, versus RSP group.

Multiple studies have revealed that the classical complement pathway is involved in microglia-mediated synapse elimination [21]. Accumulating evidence has suggested that complement proteins are critical for tagging synapses to be eliminated via microglial engulfment [49]. We hypothesized that complement C3-mediated microglial engulfment of synapses may be a crucial mechanism leading to synapse loss in the hippocampus of reserpine-treated mice. We performed high-resolution confocal microscopy by immunostaining with the presynaptic marker VGlut2, the microglia marker Iba1, and the lysosome marker CD68 (Fig. 7a). With 3D reconstruction and surface rendering using the surface function of Imaris, the internalization of the presynaptic protein VGlut2 was quantified within the lysosomal volume of each microglia in the CA1 region. The results showed that the internalization of VGlut2 in microglial lysosomes was significantly increased in reserpine-treated mice. In contrast, BoNT/A markedly reduced the VGlut2 engulfed by microglia in the RSP group (Fig. 7b). These results suggest that microglial engulfment of synapses was enhanced in reserpine-treated mice and that the increased engulfment of synapses by microglia could be suppressed with BoNT/A treatment.

### BoNT/A attenuated the reserpine-induced microglia-mediated proinflammatory response

**Fig. 8:**
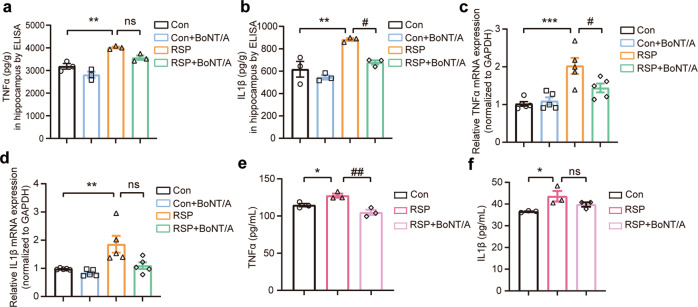
BoNT/A treatment ameliorated microglia-mediated neuroinflammation in vivo and in vitro. **a**, **b** Hippocampal TNF-α (**a**) and IL-1β (**b**) protein concentrations were measured by ELISA (*n* = 3 mice for each group. For TNF-α, *F*_(3, 8)_ = 23.78, *P* = 0.0002. For IL-1β, *F*_(3, 8)_ = 14.50, *P* = 0.0013). **c**, **d** Hippocampal TNF-α (**c**) and IL-1β (**d**) mRNA expression was determined by qRT‒PCR (*n* = 5 mice for each group. For TNF-α, *F*_(3, 16)_ = 11.79, *P* = 0.0003. For IL-1β, *F*_(3, 16)_ = 7.872, *P* = 0.0019). **e**, **f** BV2 microglial cells were exposed to 50 nM reserpine (RSP) for 24 h in the presence or absence of BoNT/A (0.1 U/mL). TNF-α (**e**) and IL-1β (**f**) in the cell media secreted by BV2 microglial cells were detected by ELISA (*n* = 3 replicates for each group. For TNF-α, *F*_(2, 6)_ = 15.95, *P* = 0.0040. For IL-1β, *F*_(2, 6)_ = 5.393, *P* = 0.0457). All data are presented as the mean ± SEM. One-way ANOVA with Tukey’s post hoc test was used. *P  <  0.05, **P  < 0.01, ***P  <  0.001, versus Con group. ^#^*P*  <  0.05, ^##^*P*  <  0.01, versus the RSP group.

To further investigate the effect of BoNT/A on the reserpine-induced microglia activation-mediated inflammatory response, we quantified the protein and mRNA levels of TNF-α and IL-1β by ELISA and qRT‒PCR. The ELISA results indicated that TNF-α and IL-1β were significantly increased by reserpine treatment, which is consistent with previous findings [50]. However, BoNT/A treatment alleviated the reserpine-induced increase in the protein levels of TNF-α and IL-1β (Fig. 8a, b). The mRNA expression levels of TNF-α and IL-1β were significantly increased in the RSP group and evidently declined in the RSP + BoNT/A group (Fig. 8c, d). To further examine the effect of BoNT/A on the microglial cell-mediated proinflammatory response, we tested the TNF-α and IL-1β cytokines in the media of BV2 cells stimulated with reserpine in the presence or absence of BoNT/A treatment. The results showed that reserpine induced significant increases in TNF-α and IL-1β cytokines in the media of BV2 cells, and the elevated TNF-α and IL-1β cytokines induced by reserpine were reversed by treatment with BoNT/A (Fig. 8e, f). Taken together, these data indicate that BoNT/A can ameliorate the reserpine-induced microglia-mediated proinflammatory response.

## Discussion

In the present study, we explored the antidepressant effect of botulinum toxin A on depression in Parkinson’s disease and the underlying mechanisms. Our findings indicated that chronic administration of reserpine could reproduce similar clinical symptoms of depression in Parkinson’s disease, including depressive and motor disorders, indicating good face validity as an animal model for the study of depression in Parkinson’s disease. Treatment with botulinum toxin A alleviated reserpine-induced depressive symptoms. BoNT/A suppressed the reserpine-induced activation of the classical complement pathway, as well as the proinflammatory factors IL-1β and TNF-α, in vivo and in vitro. BoNT/A rescued excitatory synapse loss and decreased dendritic spine density in the hippocampus of reserpine-treated mice. Moreover, BoNT/A suppressed reserpine-induced microglial activation in vivo and in vitro, thus protecting against hippocampal synapse loss by inhibiting microglial synaptic engulfment.

### Animal models of depression in PD

Modeling the depressive symptoms of PD in rodents is full of challenges. Neurotoxins causing DA neuron injury have mostly been used to establish motor symptom disturbance in studies of PD in animals. With multiple efforts, moderate lesions of DA neurons using MPTP, 6-OHDA, and rotenone have been applied to model depression in PD in most studies. However, the use of these neurotoxins still produces inconsistent results [51]. In the present study, we used a traditional mouse reserpine model of PD to investigate the underlying mechanism of action of botulinum toxin A in the treatment of depression in PD. Reserpine is reported as a specific inhibitor of the vesicular monoamine transporter that triggers an extensive depletion of DA, NA, and 5-HT [52]. The reported animal reserpine models have reproduced key features of PD symptomatology, including a wide range of motor symptoms of PD, such as akinesia, hypokinesia, limb rigidity, and oral tremor [52], as well as depressive-like and anxiety-like behaviors [53]. Previous studies have shown that acute reserpine administration at high doses ranging from 1–5 mg/kg results in the induction of hypolocomotion, catalepsy, and depressive behavioral changes (as identified in the sucrose preference test and forced swim test) in rodents [54–58]. With repeated administration at a low dose (0.1 mg/kg), chronic reserpine treatment produces deficits in emotional memory processing before the appearance of motor signs in rats [59, 60]. These studies corroborate the progressive symptoms observed in PD patients. Our studies have shown that chronic administration of reserpine induced gradual development of motor and depressive alterations. Therefore, depending on the dose and administration, reserpine is able to produce motor deficits as well as other kinds of behavioral changes, including cognitive, mood and sensory system changes, in rodents. Chronic or repeated treatment with reserpine can be a progressive animal model to study the depression prevalent in PD patients. In addition, the reserpine model also mimics key features of PD neurochemistry. A previous study illustrated that repeated administration of a low dose of reserpine decreases striatal tyrosine hydroxylase (TH) levels and reduces the number of TH^+^ cells in the substantia nigra pars compacta (SNpc) [60]. Additionally, our studies indicated the neurochemical and neuropathological alterations in a chronic reserpine-induced mouse model, accompanied by decreased DA levels in the striatum and SNpc, decreased TH striatal levels, and a reduced number of TH^+^ cells in the SNpc. Thus, our studies extended the previous findings [57] and revealed both the symptomatology and neuropathology of depression in PD. The gradual development of motor impairment induced by chronic administration of reserpine is also preceded by depressive-like symptoms and accompanied by neurochemical disturbances compatible with the pathology of PD. Depressive-like behavior was significantly alleviated in mice with reserpine-induced Parkinson’s disease treated with BoNT/A, which is consistent with a previous study showing the antidepressant-like effect of botulinum toxin A in a unilateral 6-OHDA rat model of Parkinson’s disease [35]. Thus, our data demonstrated that botulinum toxin A could ameliorate depressive-like behavior in a progressive reserpine-induced Parkinson’s disease mouse model.

### Potential role of glutamatergic neurotransmission in Parkinson’s disease with depression

The neural mechanisms underlying major depression disorders are traditionally associated with alterations in neurotransmitter systems, including 5-hydroxytryptamine (5-HT), noradrenaline (NA), and dopamine (DA), in the limbic and cortical structures. Except for the role in the regulation of mood, disturbances in these systems are involved in the pathology of PD [4]. Recently, emerging evidence has shown that the activities of glutamatergic and GABAergic neurons, with altered levels of genes associated with glutamatergic and GABAergic pathways, are involved in mice showing a depressive-like phenotype [61, 62]. However, the roles of the glutamatergic and GABAergic systems in depression in PD remain elusive. The aforementioned toxins destroy DA neurons, but the accompanying disturbances of other neurotransmitters are less studied in the limbic and cortical structures. A majority of previous studies neglect the possible contribution of non-DA neurons in the course of PD depression. In an intranigral MPTP, 6-OHDA, LPS, and rotenone model of Parkinson’s disease study, it was reported that MPTP, 6-OHDA, and rotenone, but not LPS, could induce depressive-like behaviors, predominantly associated with serotonin and dopamine [51]. In our present study, we found that excitatory VGlut2 synaptic density was decreased in the reserpine-induced PD depression model, while BoNT/A treatment could reverse glutamatergic synapse loss in the hippocampal regions. Thus, our data indicated that glutamatergic neurons were involved in the course of PD depression, and botulinum toxin A could be a novel antidepressant therapy against the glutamate neurotransmitter system for parkinsonism depression.

### Role of complement and microglia in Parkinson’s disease with depression

The structure of a brain affected by depression is quite distinct from that of a healthy brain and normally has visible manifestations of pathophysiology. Postmortem studies of parkinsonism patients with a severely depressive phenotype have revealed a decreased brain volume and a smaller brain size of the PFC and hippocampus, which are considered critical regions in depressive mood regulation. An impairment of synaptogenesis, tightly associated with synapse loss, and the atrophy of dendrites might underlie the reduction in hippocampal volume. Lower expression of synaptic-function-related genes and a loss of dendritic spines were observed in rodent models of depression [63]. Recent evidence has strongly demonstrated that complement-mediated microglial engulfment contributes to synapse loss. Synapse pruning and loss have been widely investigated during the development of neurodegenerative diseases, including PD and AD. Specifically, microglia recognize activated complement protein C3 bound to synapses tagged for synaptic elimination, triggering the microglial engulfment of tagged synapses. The decelerations or exacerbations in abnormal synaptic pruning could cause severe neural circuitry alterations, which might underlie typical neuropathological changes in neurodegenerative and neuropsychiatric disorders [20]. Synapse loss and dysfunction are observed in a region-specific manner and are strongly correlated with certain behavioral impairments related to neurological disorders, including cognitive decline and depression. However, the role of complement protein-mediated microglial synaptic engulfment in depression in Parkinson’s disease remains unknown. In our present study, we found significant activation of the classical complement pathway, with the elevation of complement proteins C1q and C3 and its associated receptor C3aR induced by reserpine in vivo and in vitro. A reduction in glutamatergic synaptic density was observed in the hippocampus of the PD depression model mice, which was rescued by treatment with botulinum toxin A. Thus, our study indicates that microglia become reactive in response to complement C1q and C3 activation and enhance the activity-dependent engulfment of synaptic materials, causing synapse loss.

In our animal model, glutamatergic synapse loss was observed in the hippocampal regions of mice with PD depression. This glutamatergic loss in PD depression may have similarities and considerable differences from those found in AD. Multiple AD animal studies have suggested the effects of neuroimmune signaling pathway dysregulation on synaptic function involving the classical complement cascade, TREM2, and ApoE [64]. The complement system activates microglia to aberrantly engulf synapses, resulting in the synapse loss observed in the hippocampus related to cognitive decline [19, 23, 65]. Additionally, abnormal complement system activation has also emerged in major depression disorders and PD [25, 44, 66]. In a repeated systemic LPS injection-induced neurodegenerative model, an increased microglial inflammatory phenotype and loss of dopaminergic neurons in the substantia nigra have been found with the involvement of the classical complement system and associated phagosome pathway [66]. Thus, ‘eat me’ signals, such as complement systems, triggering microglia-mediated synapse loss are possibly common in neurodegenerative diseases. Prefibrillar oligomeric β-amyloid (Aβ) and/or tau are related to pathological synaptic dysfunction and loss in AD. β-amyloid fibrils and apolipoprotein-E (ApoE) have been found to activate the classical complement pathway via specific binding to C1q [67, 68]. In contrast, aberrant alpha-synuclein (αSyn) aggregation has been considered a pathological hallmark of PD. Intriguingly, the complement system is involved in dopaminergic neurodegeneration in mice with α-synuclein-based PD [69]. Alpha-synuclein also activates the classical complement pathway, leading to complement-dependent cell toxicity [70]. However, whether complement mediates microglial engulfment of synapses in specific regions, such as the striatum and substantia nigra, in PD remains elusive. Although complement and microglial activation were observed in the hippocampus in our animal PD model, whether other neurotransmitters, including dopaminergic and serotoninergic neurotransmitters, are involved in hippocampal synapse loss remains to be further investigated.

Psychological stress can induce severe depressive symptoms in Parkinson’s disease [71]. Preclinical studies have shown a correlation between stress-induced changes in microglial function and the development of depressive phenotypes. As resident macrophages in the brain, ‘innate immune memory’ (IIM) in microglia has recently been demonstrated. Microglia can be preactivated or ‘primed’ by an inflammatory insult, resulting in a status of innate immune memory. The initial stimulus adapts the microglial phenotypes in a way that triggers a stronger (immune training) or weaker (immune tolerance) reaction, resulting in an enhanced or suppressed immune response to a secondary inflammatory insult [72]. With immune training and immune tolerance, microglia are reprogrammed by various inflammatory heterologous insults across short and long-time scales. Both primed and IIM microglia are pathological hallmarks of neurological diseases in rodent models, consistent with clinical observations in patients. Preclinical and clinical findings have demonstrated that communication between neurons and glial cells, especially microglia, exerts impacts on stress-induced synaptic deficits and associated behavioral consequences [73]. Specifically, complement C3-C3aR, CX3CL1-CX3CR1, CSF1-CSF1R, and purinergic signaling via P2RX7 and P2RY12 are implicated in microglia-mediated neuronal remodeling [74]. In several rodent model studies, microglial cells in the substantia nigra and hypothalamus were found to be reactive following chronic and sub-chronic stress [75, 76]. However, the critical role of microglia in Parkinson’s disease with depression is obscure. Microglia can be primed by a transient or chronic initial stimulus. Thereafter, they exhibit innate immune memory in the way of a ‘trained’ (enhanced) or ‘tolerant’ (suppressed) response to subsequent stimuli. Repeated exposure of microglia to inflammatory stimuli either exaggerates or alleviates the inflammatory response, leading to either beneficial or harmful outcomes. Previous studies have indicated that a single LPS injection (0.5 mg/kg) gave rise to immune training, while repeated LPS administration of the same dose for four consecutive days produced robust immune tolerance [77]. In another study, repeated systemic injection of mice with bacterial LPS (1.0 mg/kg) over four consecutive days induced the activation of the classical complement system and escalated microglial inflammation. The microglia-associated phagosome pathway is involved in the complement-mediated loss of dopaminergic neurons in the substantia nigra [66]. In our study, we confirmed microglial activation in the hippocampal region following chronic reserpine administration. We found that microglial activation was correlated with the classical complement pathway rather than the chemokine CX3CL1-CX3CR1-dependent pathway. In addition, microglia respond to stress-induced neuroinflammation via the secretion of cytokines, chemokines, and growth factors in the etiology of depression [78]. The pro-inflammatory cytokines IL-1β and TNF-α were significantly increased in the reserpine-induced model of PD depression, consistent with previous studies [50, 79]. Furthermore, botulinum toxin A treatment significantly reversed the abnormal microglial activation and microglia-mediated proinflammatory state. Our studies indicate that microglia prime and enhance phagocytic responses in a chronic reserpine-induced PD depression mouse model. The influence of the dose, timing, and spacing of various priming or desensitizing stimuli in mouse models of brain pathology remains to be further investigated.

### Potential roles of reserpine in neuroinflammation

Reserpine is an irreversible inhibitor of vesicular monoamine transporter 2 (VMAT2), resulting in the accumulation of neurotoxic dopamine oxidation byproducts [59]. Reserpine functions as a monoamine-depleting agent in motor activity, resulting in transient hypolocomotion and muscular rigidity. The administration of reserpine to rodents has also been suggested as a pharmacological model of depression [41, 50]. Aside from the changes in monoamine neurotransmitters upon reserpine treatment, the inflammatory response is altered in reserpine-treated animal models [50, 80]. Reserpine was found to provoke the activation of the NF-κB pathway [81], an increase in inflammatory cytokines (IL-1β, IL-6, and TNF-α) [50, 80], and microglial activation [82]. In the present study, reserpine was found not only to increase proinflammatory cytokines (IL-1β and TNF-α) but also to provoke the activation of microglia. However, further investigations are needed to determine the underlying mechanisms of action of reserpine in neuroinflammation.

### Potential mechanisms of action of BoNT/A in treating neuroinflammation

BoNT/A undergoes binding to nerve terminals, internalization within the lumen of the SV, and translocation of the L chain into the cytosol, thus cleaving SNAREs. During this process, BoNT/A can bind to two independent receptors on the presynaptic membrane, a polysialoganglioside (PSG) and the glycosylated SV2C protein, to facilitate internalization. By interacting with SNAP25, BoNT/A blocks the release of neurotransmitters and neuropeptides by vesicle-regulated exocytosis [83]. BoNT/A affects not only neuronal functions but also microglial activation. It has been suggested that TLR2 is an important molecular target for BoNT/A [84]. BoNT/A exerts its anti-inflammatory action via its receptor to inhibit the NF-κB, p38, and ERK1/2 signaling pathways in microglia [84, 85]. TLR2 is suggested to regulate complement activation, and complement C3 promotes NF-κB activation in a TLR2-dependent manner [86, 87]. It has been reported that microglia are the main source of complement C1q [88]. Thus, BoNT/A may have an impact on the activation of complement and microglia via interaction with receptors on microglia, thus inhibiting microglia-mediated neuroinflammation.

We acknowledge some limitations in our current study. First, our study was mainly based on investigations in male mice only. Many studies have found that antidepressant therapies in the female mouse models of depression are affected by the estrus cycle and estrogen [89]. Second, synapse loss and deficits in synaptic plasticity associated with depression are broadly distributed throughout the central nervous system, including the medial prefrontal cortex (mPFC), hippocampus, and amygdala. Given the postmortem studies of structural alterations in the hippocampus, we mainly focused on structural synaptic plasticity in this study. Overall, studies including female subjects and other brain regions are warranted to verify the generalizability of our findings across sexes and brain areas.

Notwithstanding these limitations, our findings demonstrated that botulinum toxin A could alleviate depressive-like behavior in a reserpine-induced mouse model of depression in Parkinson’s disease via complement pathway-mediated microglia engulfment and microglia-elicited neuroinflammation. Our work also revealed the alteration of glutamatergic synapses in Parkinson’s disease with depression, indicating that glutamatergic neurotransmission is a promising therapeutic strategy for depression disorders in Parkinson’s disease.

## Supplementary information

Figure S1

Figure S2

Supplementary data
