# Supplementary figures and images for: Botulinum neurotoxin A ameliorates depressive-like behavior in a reserpine-induced Parkinson’s disease mouse model via suppressing hippocampal microglial engulfment and neuroinflammation
Source: Acta Pharmacol Sin. 2023 Feb 10;44(7):1322–36. doi: 10.1038/s41401-023-01058-x (PMC10310724; doi:10.1038/s41401-023-01058-x)

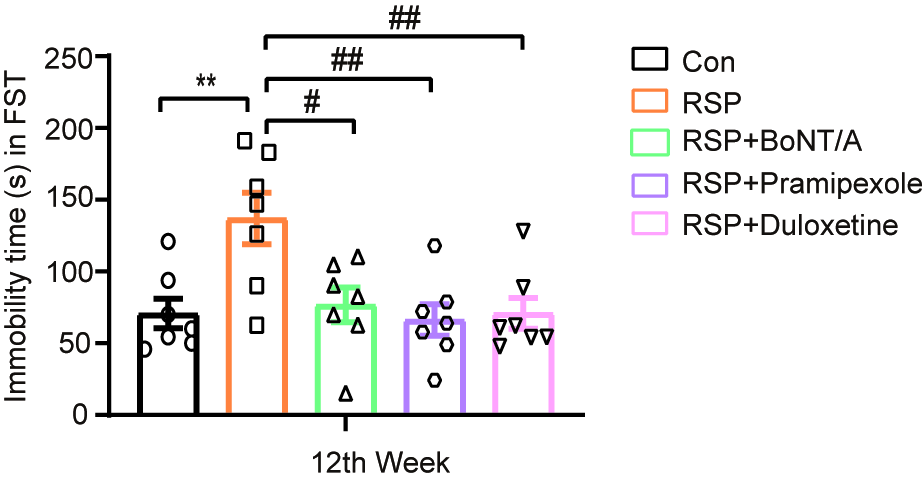

Supplement: Supplementary file 1 — Figure S1 [file 41401_2023_1058_MOESM1_ESM.tif]

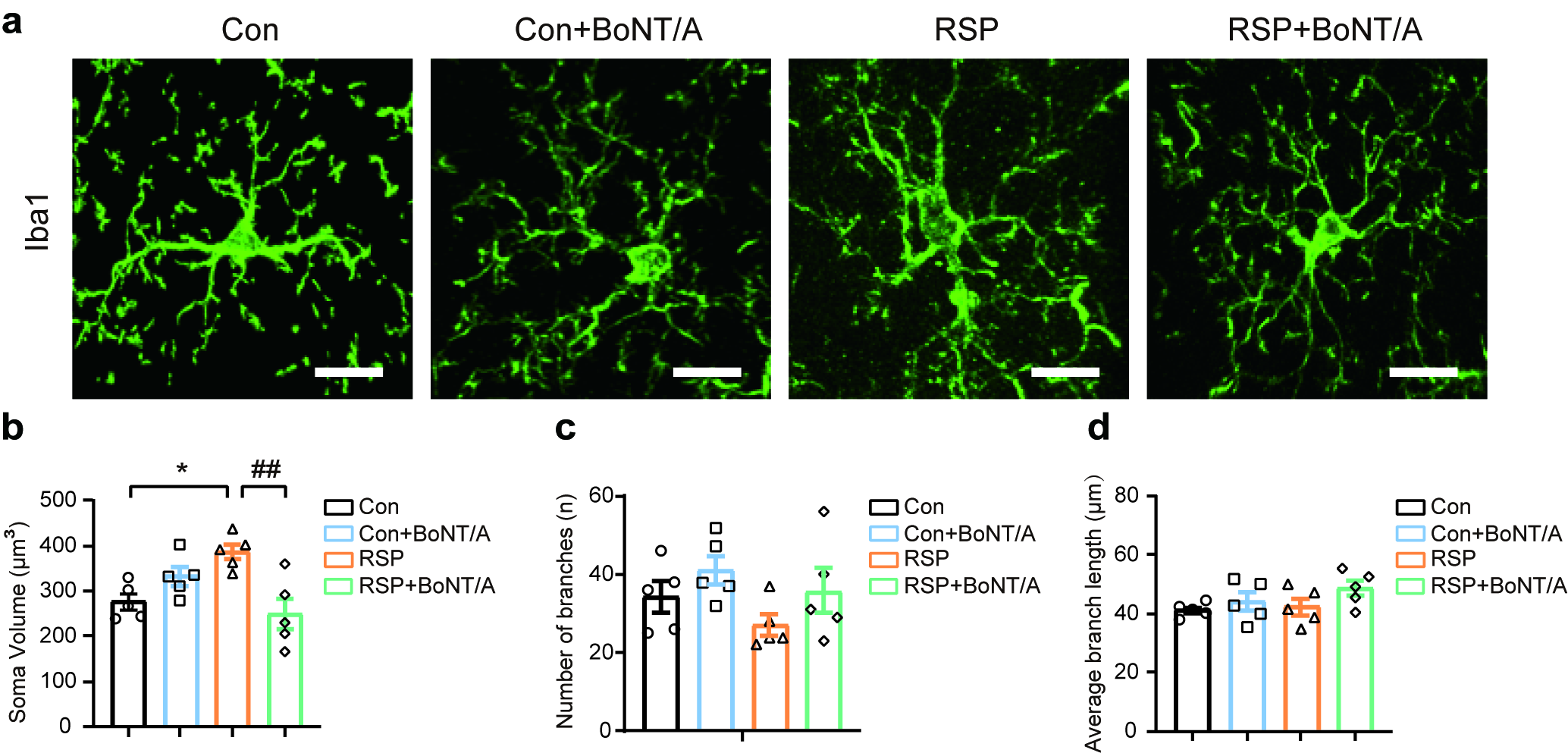

Supplement: Supplementary file 2 — Figure S2 [file 41401_2023_1058_MOESM2_ESM.tif]
