# Supplementary material for: Botulinum neurotoxin A ameliorates depressive-like behavior in a reserpine-induced Parkinson’s disease mouse model via suppressing hippocampal microglial engulfment and neuroinflammation
Source: Acta Pharmacol Sin. 2023 Feb 10;44(7):1322–36. doi: 10.1038/s41401-023-01058-x (PMC10310724; doi:10.1038/s41401-023-01058-x)
Supplement: Supplementary file 3 — Supplementary data [file 41401_2023_1058_MOESM3_ESM.docx]

**Supplementary data:**

Fig. S1: Effect of BoNT/A and other positive control drugs on the performance of reserpine-treated mice in the FST. The number of mice used in all groups was 7. All data are presented as the mean ± SEM. One-way ANOVA with Tukey’s post hoc test was used. **P < 0.01, versus Con group. ^#^P < 0.05, ^##^P < 0.01, versus the RSP group.

Fig. S2: Effect of BoNT/A on microglial morphological changes. (a) Representative images used for the morphological characterization of microglia. Scale bar = 10 μm. (b) Quantification of microglial soma volume based on 3D surface rendering. (c) Quantification of the number of branching points of microglial processes by skeletonization analysis. (d) Quantification of the average branch length of microglial processes with the skeletonization method. n = 5 images from 3 mice for each group. All data are presented as the mean ± SEM. One-way ANOVA with Tukey’s post hoc test was used. *P < 0.05, versus Con group. ^##^P < 0.01 versus the RSP group.
